# Gastrointestinal Symptoms and Feeding Problems and Their Associations with Dietary Interventions, Food Supplement Use, and Behavioral Characteristics in a Sample of Children and Adolescents with Autism Spectrum Disorders

**DOI:** 10.3390/ijerph17176372

**Published:** 2020-09-01

**Authors:** Katarina Babinska, Hana Celusakova, Ivan Belica, Zofia Szapuova, Iveta Waczulikova, Dagmar Nemcsicsova, Aleksandra Tomova, Daniela Ostatnikova

**Affiliations:** 1 Academic Research Centre for Autism, Institute of Physiology, Faculty of Medicine in Bratislava, Comenius University, 813 72 Bratislava, Slovakia; hana.celusak@gmail.com (H.C.); ivan.belica@fmed.uniba.sk (I.B.); szapuova.z@gmail.com (Z.S.); nemcsicsova@assiduo.sk (D.N.); aleksandra.tomova@fmed.uniba.sk (A.T.); daniela.ostatnikova@fmed.uniba.sk (D.O.); 2 Department of Nuclear Physics and Biophysics, Faculty of Mathematics, Physics and Informatics, Comenius University, 842 48 Bratislava, Slovakia; waczulikova@gmail.com; 3 Gastroenterology Centre ASSIDUO, 811 08 Bratislava, Slovakia

**Keywords:** autism spectrum disorders, gastrointestinal problems, food selectivity, dietary interventions, food supplements, behavior

## Abstract

Autism spectrum disorders (ASD) are characterized by impairments in social interaction, communication, and restricted, stereotyped behavior. Gastrointestinal (GI), nutritional, and feeding problems are often reported in ASD. We investigated the prevalence of GI symptoms, food selectivity, and mealtime difficulties, and their associations with dietary interventions, food supplement use, and behavioral characteristics in a sample involving 247 participants with ASD and 267 controls aged 2–18 years. Data were collected by a questionnaire. GI symptoms were observed in 88.9% of children and adolescents with ASD, more often in girls than in boys. High rates of food selectivity (69.1%) and mealtime problems (64.3%) were found. Food supplements were used by 66.7% of individuals, mainly vitamins/minerals, probiotics, and omega-3 fatty acids. In the ASD sample, 21.2% of subjects followed a diet, mostly based on gluten and milk restriction, including individuals exhibiting food selectivity. Frequency of GI symptoms, food selectivity, and mealtime problems correlated weakly, but significantly with behavioral characteristics in the ASD group, but not with food supplement use. The study demonstrated that higher frequency of GI symptoms, food selectivity, and mealtime problems are a common problem in pre-schoolers, schoolchildren, and adolescents with ASD, and together with dietary modification, they are significantly associated with ASD.

## 1. Introduction

Autism spectrum disorders (ASD) are lifelong neurodevelopmental disorders characterized by the core symptoms that include impairments of social communication, social interaction, and restricted or repetitive patterns of behavior, interests, and activities [1]. The term “spectrum” refers to heterogeneity in the clinical manifestation and severity of ASD symptoms, as well as intellectual capacity, daily adaptive functioning, or other phenotypic characteristics [2]. The phenotypic heterogeneity seems to reflect etiological heterogeneity; according to current concepts, ASD are a multifactorial disease with polygenic inheritance, with epigenetic and environmental factors involved in its pathomechanisms. Several hypotheses have been suggested to explain pathophysiology of ASD, but despite advances in understanding of the disorders, exact mechanisms remain unclear [3,4]. A growing trend in prevalence rates of ASD is observed [5]. In USA, the prevalence increased from 1 in 150 children in 2000 to 1 in 54 children in 2016, i.e., the disorders affect approximately 1.7% of children, of that 4.3 times more boys than girls [6]. Most individuals diagnosed with ASD in childhood retain their diagnosis until adulthood [7]. Due to severity of symptoms, increasing prevalence, unclear etiopathogenesis, and absence of causal therapy, ASD represent a serious public health issue [8].

ASD is associated with a large variety of comorbid conditions that occur in individuals with ASD more frequently than in subjects with typical development [3,9]. Gastrointestinal (GI) dysfunction is one of the most frequent problems, GI symptoms, such as constipation, diarrhea, abdominal pain and bloating, or gastroesophageal reflux have been reported in 23–70% of individuals with ASD. A greater than threefold higher risk of constipation and diarrhea between children with and without ASD and a greater than twofold elevated risk of abdominal pain have been found [10]. It was shown that severity of GI problems was associated with severity of behavioral symptoms of ASD [11,12,13]. GI disorders may have atypical presentation in ASD, namely as exacerbation of challenging behavior without a clear environmental trigger, especially in children who exhibit limited verbal communication [14].

High prevalence of feeding problems and unusual eating behaviors, partly associated with GI problems, have been reported in ASD [15,16,17]. A meta-analysis revealed that a child with ASD is five times more likely to exhibit feeding difficulties than a child without ASD is [18]. The commonly identified issues include food selectivity, defined as eating only a narrow variety of foods, as well as aggression, or tantrums, during mealtime, or different eating rituals and stereotypies [15,18,19]. In children with feeding difficulties, more severe ASD symptoms were observed than in children free of feeding problems [20].

Evidence shows high rates of dietary interventions in children and adolescents with ASD [20,21]. In some instances, a child’s diet is modified due to medical conditions, such as gastrointestinal symptoms or food allergies [22]. However, nutritional interventions in subjects with ASD are commonly introduced as an alternative treatment method of behavioral symptoms. Elimination diets are believed to reduce the deleterious effects caused by the improper metabolism of some food substances contributing to symptoms of ASD [23]. At present time there is no cure for ASD, and the available interventions for improving core symptoms are limited and often not satisfactory [3,24]. Different types of nutritional dietary approaches in ASD are reported, such as gluten-free and casein-free (GFCF) diet; the gut and psychology syndrome (GAPS) diet, ketogenic or specific carbohydrate diets. Popular food supplements include probiotics, omega-3 polyunsaturated fatty acids (PUFAs), and vitamins A, C, B6, and B12; magnesium or folate [25]. There is limited information about associations of GI and feeding problems with dietary interventions and supplement use.

There is evidence that GI and feeding problems are associated with behavior of children and adolescents with ASD [11,15]. They may have adverse effects on their functioning and clinical management [3]. Similarly, feeding problems and dietary imbalances may result in impairments of nutritional and health status of the individuals [18]. Thus, identification and treatment of these conditions needs to be emphasized. Despite growing evidence, GI, nutritional and feeding problems, food selectivity, and related conditions in people with ASD, as well as their associations with behavioral presentations are not fully explained and require additional studies.

The aim of the study was to assess in a sample of children and adolescents with ASD, the prevalence and types of GI symptoms, frequency of food selectivity and mealtime problems. We also investigated their associations with dietary interventions and supplement use, as well as the prevalence and types of diets and dietary supplements used. Finally, we analyzed whether GI problems, feeding and diet-related factors are correlated with ASD severity and behavioral characteristics of the individuals.

## 2. Materials and Methods

### 2.1. Sample

The study was performed by the Academic Research Centre for Autism (ARCA) at Faculty of Medicine, Comenius University, Bratislava, Slovak Republic, data were collected over the period 2013–2019. The sample involved 247 children and adolescents with ASD aged 2–17 years; predominance of boys (86.6%) corresponds with the higher prevalence rates of ASD in boys than in girls. In the sample, children aged 2–5 years prevail (55.6%), as majority of the individuals were ASD cases newly diagnosed in ARCA. The sample included also participants recruited via special schools for children and adolescents with ASD. Inclusion criteria involved diagnosis of ASD and age range 2–18 y. Exclusion criteria included severe sensory or motor impairments, and suspected psychotic condition.

Control sample comprised 267 individuals of the same age with typical neurodevelopment (neurotypical) free of ASD, including siblings of children with ASD (*n* = 46) as well as non-related individuals (*n* = 221) recruited in kindergartens and schools, or via on-line invitation to participate in the study. Absence of ASD symptoms or diagnosis of a neurodevelopmental disorder was based on parents’ reports. Table 1 displays the characteristics of the sample.

### 2.2. ASD Diagnosis

Study participants were diagnosed with ASD by trained examiners. The diagnostic tools involved the Autism Diagnostic Interview-Revised (ADI-R), and the Autism Diagnostic Observation Schedule—Second Edition (ADOS-2), both considered as an international gold standard in the ASD diagnostic process. ADI-R is a semi-structured interview with parents or caregivers that concerns development and current condition of a child. It covers three domains of symptomatology according to ICD-10: (A) social interaction, (B) social communication, and (C) repetitive and restricted behavior and interests. Each domain provides a cut-off score to meet ASD criteria and allows evaluation of symptom severity [26].

ADOS-2 is a protocol consisting of structured and semi-structured tasks involving interaction between the examiner and examined person [27]. ADOS-2 has five versions (modules) based on the level of expressive language and the age of the examinee, our sample with ASD included individuals examined with modules 1, 2, 3, and the Toddler module. The output score of the examination was divided into two domains in accordance with DSM-5 criteria: (A) Social Affect (SA) that includes social behavior and communication and (B) Restricted and Repetitive Behaviors (RRB). Total raw score was determined that provides a cut-off for ASD classification and allows to evaluate symptom severity for comparing children in the same module. Calibrated severity scores (CSS) with values ranging 1–10 were calculated allowing comparison of symptom severity across different modules [28,29,30]. Consistent with the standard ADOS/ADI-R scoring, all scores of 3 were converted to 2, such that the range for each item was 0–2.

ASD diagnosis was made based on clinical evaluation; all children enrolled in the study had to meet criteria for ASD on both ASD diagnostic tools. The sample involved predominantly individuals with moderate or severe symptoms based on ADOS-2 symptom calibrated severity scale. Individuals were classified as verbal (capable to use functional expressive speech) or non-verbal according to ADI-R criteria. Behavioral characteristics of the sample with ASD based on the ADI-R and ADOS-2 scores are presented in Table 2.

### 2.3. Data Collection

Data concerning GI problems, mealtime behavior, and dietary interventions were collected by self-reported questionnaires, designed for the purpose of the study. They were completed by a child’s parents/caregivers, or by adult control individuals themselves.

For assessment of GI symptoms, adaptation of GI severity index questionnaire was used [31]. Presence of six GI symptoms in last 3 months was assessed: abdominal pain, bloating, constipation (defecation less than two times/week)/hard stools (normal frequency of defecation), diarrhea (loose stools three times per day or more)/loose stools (loose consistence but normal frequency of defecation), pain during defecation, and voluminous stools.

Frequency of food selectivity—refusal to consume a variety of food was assessed, as well as mealtime problems, including anger, crying, or screaming during mealtime, requesting to have the food prepared in the same way, and self-injurious behavior during meal. In a part of the ASD sample, data on aggressive behaviors during meal were collected, however, not in the control sample.

Data on frequency of GI symptoms, food selectivity, and mealtime problems were rated on a 5-point Likert scale: never (0), 1–3 times per month (1), once a week (2), several times per week (3), and daily (4). For analysis and presentation of the prevalence rates, the data were grouped into three categories: never (0), rarely: 1–4 times per month (1 and 2), frequently: several times per week or daily (3 and 4).

Information about usual diet of study individuals was collected. In cases when parents restricted/modified the child’s diet by excluding some food items or food groups, the child was ranked into category “dietary restriction”. The parents were asked to specify the type of dietary modification, and to indicate if the diet was prescribed by a medical doctor (e.g., due to a medical condition), or if it was based only upon a decision of the parents.

We collected data concerning food supplement use in recent 12 months. All dietary supplements used by a child were recorded, together with the information how many weeks/months the child was using each supplement. Vitamin D was excluded from the analyses, because prophylactic use of this vitamin is commonly advised by pediatricians, due to generally low plasma vitamin D levels of the population in Slovakia [32]. Similarly, a short-term use of probiotics during or following a regimen of antibiotics that is routinely recommended by clinicians, was not considered in data analyses.

### 2.4. Statistical Analysis

The demographic and clinical characteristics were analyzed using descriptive and inferential statistics. Two-sample *t*-test or alternatively Mann–Whitney test was used to test for between-group differences. Categorical variables are presented as counts and relative frequencies. Differences between categorical variables were tested by chi-square test (χ^2^) or by Fisher’s exact test, when appropriate. The Spearman’s rank correlation coefficients were used to assess the associations between selected variables. Multivariable logistic regression analysis was performed to evaluate the relationship between a binary (dichotomous) variable (grouping variable) and a predictor variable or variables. They included frequency of GI symptoms (range 0–4), frequency of food selectivity (range 0–4), frequency of mealtime problems (range 0–4), dietary intervention (yes = 1, no = 0), and supplement use (yes = 1, no = 0) when accounting for control variables age (years) and gender (M = 1, F = 0). All tests were conducted at a significance level of 5% using StatsDirect 3.0.191 software (Stats Direct Ltd., Cheshire, UK), Statistica 13.1 software (TIBCO Software Inc., Palo Alto, California, USA), and GraphPad Prism 8.0 (GraphPad Software, Inc., San Diego, California, USA).

### 2.5. Ethical Statement

Written informed consent was obtained from all subjects or their caregivers. The Ethics Committee of the Comenius University Faculty of Medicine and University Hospital in Bratislava approved the study protocol. The study conformed to the code of ethics stated in the Declaration of Helsinki.

## 3. Results

### 3.1. Gastrointestinal Symptoms

We found that 88.7% of subjects with ASD and 79.0% of controls experienced gastrointestinal symptoms in last 3 months (χ^2^ = 8.7, *p* = 0.003). Individuals with ASD exhibited GI symptoms more frequently than the controls (χ^2^ for trend = 28.9, *p* = 0.000, Table 3). In the group with ASD, 47.6% of individuals suffered from GI symptoms several times per week or daily in contrast to 24.3% of controls, whereas in the control group less frequent occurrence of GI symptoms (1–4 times/month) prevailed (41.1% of ASD, 54.7% of controls). GI symptoms occurred in girls with ASD more commonly than in boys (97.1% vs. 87.6%, χ^2^ = 13.57, *p* = 0.009), of that 70.6% of girls but only 44.5% of boys exhibited problems several times per week or daily. No gender differences were found in the control group. No significant differences in frequency of GI symptoms were observed across the age groups 2–5, 6–10, and 11–18 y old children and adolescents, neither in the sample with ASD nor in the controls. Constipation/hard stool consistency was the most frequent GI symptom, that occurred much more commonly in the sample with ASD than in controls (61.9% vs. 44.6%, χ^2^ = 15.5, *p* = 0.000). Similarly, higher prevalence of voluminous stools was found in ASD, (51.0% vs. 22.1% χ^2^ = 46.6, *p* = 0.000), frequency of other symptoms did not significantly differ between the groups. Individuals with ASD displayed higher degree of cumulating of more GI symptoms than the controls (χ^2^ for trend = 9.32, *p* = 0.002).

### 3.2. Food Selectivity and Mealtime Problems

In the sample with ASD, high prevalence of food selectivity (69.1%) and mealtime problems (64.3%) was found in comparison to the controls (37.1% and 25.9%, respectively, *p* = 0.000, Table 4). A large proportion of individuals with ASD displayed food selectivity several times per week or on a daily basis (48.8% vs. 13.1% of controls), similarly the mealtime problems (41.8%), while in the controls they occurred with much lower frequency. In the study, we assessed four types of mealtime problems: anger, requesting food to be prepared in the same way, crying and shouting during meal, self-injurious behavior. All of them were significantly more prevalent in ASD. In addition, in the ASD group, 11.4% of subjects exhibited aggression during mealtime; but we have not assessed this behavior in the control group.

In both ASD and control group, no correlations of food selectivity and mealtime problems with age or gender were found. In ASD, the frequency of GI symptoms weakly, but significantly correlated with frequency of both selective behavior (ρ = 0.216; *p* = 0.001) and mealtime problems (ρ = 0.180, *p* = 0.005). Mealtime problems significantly correlated with food selectivity (ρ = 0.531, *p* = 0.000). Table with correlations is presented in Section 3.5.

### 3.3. Dietary Interventions

In the sample with ASD, 21.2% of subjects followed a diet in last 3 months (Table 5). The GFCF (gluten-free/casein-free) diet based on elimination of both gluten and casein was the most prevalent type of diet; it was followed by 40.4% of all children and adolescents on a diet. Part of the sample consumed diet that was free or restricted only in gluten (17.3%), casein (5.8%), lactose, or milk (23.1%). Gut and Psychology Syndrome (GAPS) diet was followed by 15.4% of individuals with ASD. Other foods that were restricted by smaller numbers of individuals (30.8%) included items such as eggs, sugar, or histamine-containing foods. Within the time period of 3 months that was considered in our study, some individuals switched from one type to another type of diet.

The parents were asked to indicate the reasons for putting their child on a diet. Of 52 children who followed a diet, in 18 cases (34.6%) they reported that a medical doctor due to a medical condition, such as celiac disease, milk allergy, or other allergies or intolerances, prescribed the elimination diet. In 29 cases (55.8%), the parents decided for the diet even though the child did not display symptoms of a disease that requires dietary treatment. Some of them decided for the diet because they studied resources encouraging that this might be treatment for ASD, others followed the diet because they observed improvements of behavior and/or GI symptoms. The GFCF diet was introduced also due to elevated levels of opioid peptides detected in the children by previous urianalysis.

No significant correlation between following a diet and severity of GI symptoms or mealtime problems was found. We observed a weak significant negative correlation between dietary interventions and frequency of food selectivity, i.e., “picky eaters” were slightly less likely to be on a diet (correlations are presented in Section 3.5). However, a further analysis revealed that of all children who followed a diet, more than one third (36.5%) exhibited food selectivity several times per week or daily.

In the control sample, only 13 individuals (4.9%) followed a diet. Of that, in 10 cases the parents reported that it was recommended by a pediatrician due to medical conditions, and the remaining children have not provided the cause for a diet.

### 3.4. Dietary Supplement Use

There was no significant difference in the rates of dietary supplement use between the sample with ASD and controls (Table 6). In both groups, high rates of intake of multivitamins and/or multiminerals were observed, but in respect to use of other supplements, significant differences between ASD and control group were found. In the ASD group, use of probiotics was significantly more prevalent (42.8% of subjects), as well as of omega-3 PUFAs (28.3%), carnosine and vitamin B (both 12.7%). In the control sample, high rates of vitamin C use were found (40.7%), followed by probiotics (32.0%) and imunoglukan (biologically active polysaccharides for immunity boosting, 19.2%). In both ASD and control group, the supplement users consumed on average two types of supplements in the last 12 months. Children who were following a diet were more likely to use also food supplements (correlations are presented in Section 3.5).

Multivariable analyses were performed using the information provided by the bivariate analysis, with the aim of adjusting the model for some confounding variables. By adjusting the model by age and gender, it was observed that all but one predictor were significantly associated with ASD (Table 7). Higher frequency of GI symptoms, food selectivity, and mealtime problems, together with dietary modification without supplements significantly increased odds of being classified as ASD case (Deviance chi-square = 224.04; *p* < 0.001).

Since the gender distribution between the sample with ASD and controls significantly differed in the naïve analysis, we performed a stratified analysis to examine the potential role of gender on the relationship between each explanatory variable and ASD. Magnitudes of the moderating effects with the categorized factors could be depicted as so called forest plots that identified gender as a modifier of the strength of association between GI symptoms and ASD. Therefore, we conducted data-driven regression analyses where the effect modifier interaction terms were added in the original model (Table 7) as additional explanatory variables one at a time. The results of multivariable modelling indicated a likely interaction between gender and GI problems (OR_gender x GI_ = 0.662; 95%CI: 0.42–1.05; *p* = 0.082). Thus, having a higher GI score may be associated with increased odds of being classified as ASD in girls (coded as 0), but with lower odds in boys (coded as 1). No interactions of gender with food selectivity, mealtime problems, diet, and food supplement use were found. Assuming that the interaction was not specified a priori, and that interaction terms adversely affect parsimony of the model and may lead to problems associated with sample size, we consider this finding as preliminary.

### 3.5. Correlations with Behavioral Factors in Children with ASD

We further explored pairwise linear correlations (Table 8) focused on the strength of association between GI symptoms, food selectivity, mealtime problems, dietary intervention, food supplement use, and behavioral characteristics of the children with ASD. Behavioral markers included the main domains of ADI-R and ADOS-2 diagnostic tools, representing the main characteristics of ASD, as well as the ability to use spoken language for communication (verbal vs. nonverbal).

Weak, but statistically significant positive correlations were found between frequency of GI symptoms, food selectivity, and severity of the core symptoms of ASD based on ADI-R scores. Similarly, mealtime problems positively correlated with ADI-R score in qualitative abnormalities in communication, restricted, repetitive, and stereotyped patterns of behavior (ADI-R). No correlation between GI symptoms and ASD severity based on ADOS-2 scores, with exception of a weak negative correlation with restricted, repetitive, and stereotyped patterns of behavior. We explain this phenomenon by methodological differences of ADOS-2 and ADI-R, while ADOS-S is a short-lasting interaction of the individual by the examiner, while ADI-R is based on an extensive interview covering a longer period of development of the child with ASD.

We have not found any significant correlations between dietary interventions or food supplement use with behavioral characteristics based on ADOS-2 or ADI-R scores in the main domains. Verbal skills were negatively correlated with dietary intervention (ρ = −0.169, *p* = 0.009), and there was a trend towards negative correlation with food supplement use (ρ = −0,120, *p* = 0.062), i.e., non-verbal children were more likely to be on a diet or to use food supplements, on the other hand, they were less likely to display food selectivity and mealtime problems.

Correlations between GI symptoms, food selectivity, mealtime behavior, dietary interventions, and supplement use are explained in the previous sections.

## 4. Discussion

The study investigated multiple aspects of GI dysfunction and feeding issues in a sample of children and adolescents with ASD, as well as their associations. Results show that GI symptoms, food selectivity, mealtime problems are prevalent across all age-categories of children and adolescents. In comparison to behavioral or other clinical concerns associated with ASD, these problems are often getting insufficient attention of health professionals and caregivers of individuals with ASD [3]. In the following text, we would like to discuss mainly the public health aspects of our findings, and to point at their possible pathomechanisms and connections with the core symptoms of ASD.

### 4.1. GI Symptoms

In accordance with other studies, we showed a high prevalence of GI symptoms in children with ASD that persisted across all age groups. Individuals with ASD were more severely affected by GI problems than the controls: almost half of the children and adolescents with ASD suffered from GI symptoms several times per week or daily, in addition, subjects with ASD tended to present more GI problems than the controls. The main complaint was constipation and/or hard stools, which is in agreement with findings of other studies [14,15,33,34].

We demonstrated that higher frequency of GI symptoms was associated with higher scores in all ADI-R domains, i.e., with more severe presentation of ASD symptomatology: social interaction, social communication, and repetitive and restricted behavior and interests. These results are similar to other studies that have shown more severe ASD core symptoms in children with GI problems than in children without GI problems [11,35]. Alterations of behavior, such as aggression, self-injurious activity, or sleep problems are often misleadingly attributed to be just “symptom of the autism” [14], but it is possible that behavioral impairments in ASD are exacerbated or even partially due to the underlying gastrointestinal problems [11]. Additionally, the evidence linking GI dysfunction with behavioral presentations of ASD may indicate that they share some common pathomechanisms [36].

The existence of a GI pathology specific to ASD has not been established, and mechanisms of GI disorders in ASD are not fully explained. GI symptoms seem to have multifactorial basis [37]. Recently, much attention is focused on the microbiota residing in the GI system [38]. Evidence shows that gut microbiota and its metabolites influence the brain via the gut–brain axis, i.e., a bidirectional communication between the gut and the brain through neural, endocrine, and immune mechanisms. In ASD, differences in abundance and composition of gut microbiota have been observed [39], and the altered signaling from gut to the brain has been suggested as a potential contributor to the development of ASD and its behavioral presentations. At the same time, altered gut microbiota may contribute to the GI problems associated with ASD [36].

There is growing evidence of gender differences in ASD symptoms [40]. In our study, girls with ASD exhibited GI problems more frequently than boys; 70.6% of girls but only 44.5% of boys had problems several times per week or daily. As found on bivariate analysis stratified by gender, among boys, those who scored by one unit more on the Likert scale of GI symptoms were, on average, at 1.39 times higher risk of being classified with ASD, compared to those in the reference GI category (free of GI problems). In girls, an increase by one unit on the Likert scale of GI symptoms was associated with a 2.55 times higher risk of having an ASD, compared to those in the reference GI category. Modifying effect of gender on this relationship was also confirmed in the multivariable analysis. This finding suggests that presence and severity of GI symptoms may be a supplementary biomarker in diagnosing ASD in girls. More investigation is needed for explanation of this observation. Kushak and Winter [41] suggested that intestinal microbiota that is believed to interact with sex hormones is a factor associated with higher ASD prevalence in boys. Thus, we may hypothesize that gut microbiota might contribute also to gender differences in GI symptoms. Still, due to limited number of girls in our sample, this finding needs to be interpreted with caution.

### 4.2. Food Selectivity and Mealtime Problems

Food selectivity resulting in imbalances in the diet composition may be a factor contributing to GI problems, at least in a proportion of individuals with ASD [12]. In our study, significant correlation between severity of GI symptoms with food selectivity and mealtime problems was found. Similarly, in our earlier survey, children with ASD who were food selective, had more GI symptoms compared to children free of food selectivity [42].

About 70% of children with ASD are reported to have some feeding problems [17]. Besides the “picky eating”, other undesirable mealtime behaviors observed in ASD, such as adherence to routines, resistance to new or non-preferred foods, or tantrums have been shown to adversely influence the diet composition of many children with ASD [17]. In our sample with ASD, high rates of problem behavior related to food intake were demonstrated in boys and girls of all ages that correlated with severity of ASD symptoms in ADI-R domains. There is evidence that feeding problems in ASD are of multifactorial origin, and they appear to be associated with the core behavioral characteristics of ASD. They reflect repetitiveness and preference for sameness, ritualism, unusual interest in sensory properties of food, but also diminished responsiveness to social reward, and increased reactivity in response to frustration [17,43].

To some extent, food selectivity and feeding disorders are present in neurotypical children, but they are more severe in ASD [44]. A study revealed that in ASD, the “picky eating” appears at early age and escalates more quickly than in typically developing children [43]. The food aversion does not resolve over time as the child develops, therefore it is not recommended to wait for the difficulties to disappear or that the child will “grow out of the problem” [45,46].

The most frequently omitted food group is vegetables, followed by fruits [16,42]. Selective children with ASD often prefer foods with low nutritional value and high in fat, salt, and sugar [19]. Children with ASD were shown to have a lower protein intake, and their diets may be low in micronutrients [47,48]. Food variety has been shown to be a predictor of nutritional status of children with ASD [49]. Mealtime problems and unusual dietary patterns are one of the factors contributing not only to undernutrition, but also to higher risk of obesity in children with ASD [50].

Feeding problems in ASD may remain unattended by the healthcare providers. This is because selective eating patterns are not necessarily associated with higher risk for growth retardation that is a marker of nutritional deficits and triggers clinical attention of pediatricians [18].

### 4.3. Dietary Interventions

There is evidence that individuals with ASD are more frequently affected by food allergies and intolerances, requiring diet as a treatment method of their medical condition [22]. In our study, more than 20% of children and adolescents were on a diet, of that about one third reported that they followed a diet on recommendation of their pediatrician. However, more than 50% of children and adolescents were put on a diet, although they did not suffer from a medical condition that is typically treated with a diet. We have not found a correlation between GI problems and diets, thus it does not seem that GI problems were a significant cause for putting the child on a diet. Evidence shows that in a sizeable proportion of individuals with ASD, the dietary intervention is a form of complementary and alternative medicine, i.e., practices and products that are not part of conventional Western medical care [51]. Implementation of a GFCF (gluten-free/casein-free) diet is the most used dietary intervention in ASD [23,52]. It is based on the “opioid excess hypothesis”. It postulates that ASD results from a metabolic disorder in which opioid peptides produced by metabolism of gluten and casein pass through an abnormally permeable intestinal wall into blood, and exert harmful effects on processes in the brain [52]. Although scientific evidence shows some improvements in behavior on GFCF diet, there is insufficient data to support its broader use [23,53]. Several caregivers of our ASD subjects reported that they rather reduced than fully eliminated either gluten or casein, which raises questions about efficacy of such “partial” dietary treatment.

GAPS (Gut and Psychology Syndrome) diet is a strict elimination diet that recommends to cut out foods such as grains, pasteurized dairy, starchy vegetables, or refined carbohydrates. Although it is broadly suggested in non-scientific resources, it was very scarcely studied, thus there is lack of evidence about efficacy of this diet and doubts about its safety. Better studied is the ketogenic diet (KD) that is also based on major restriction of carbohydrates. Although the evidence is promising, data are still limited, and insufficient to encourage KD as a treatment for ASD [54].

A review of existing data shows that gluten-free diet is not an appropriate choice in individuals without a medical condition due to their lower content of energy, several vitamins and minerals and the dietary fiber, and potential heavy metal exposure [55]. Gluten free diets reduce consumption of starchy foods that are often preferred by children with ASD [52]. Children with reduced milk intake or milk-free diet face an increased risk of growth deceleration and suboptimal intake of several micronutrients, mainly calcium [52,56].

We demonstrated in our study that elimination diets were introduced also to individuals who exhibited food selectivity. There is evidence that children with ASD who present mealtime problems are at higher risk of nutrient deficiencies than their nonselective peers [16,17]. The combination of food selectivity and nutritional limitations raises important questions regarding the use and elevated risk of adverse effects of dietary interventions. As the elimination diets are associated with higher risk of nutrient deficiencies, the caregivers should employ utmost caution when deciding to pursue this form of treatment.

A meta-analysis of dietary approaches to ASD does not support non-targeted dietary interventions as treatment of ASD. Due to heterogeneity in ASD presentation, it appears that there is a potential role for some specific dietary interventions in the management of some symptoms in some individuals. There is a need for better-designed clinical trials assessing efficacy and safety of dietary interventions in ASD [21].

### 4.4. Food Supplements

High rates of food supplement use were found in our study in the ASD group, but also in the control sample. Generally, an increasing trend in food supplement use among children and adolescents has been observed [57], that are given mainly to prevent or treat illnesses [58]. A study that involved children with ASD reported that the top reasons of parents for giving supplements were also to enhance the child’s diet, and to promote immune system function [51]. Evidence shows that the most frequently used forms of supplements are vitamins and minerals in a variety of combinations; also in the ASD population [47,51,57]. Interestingly, there are reports that supplements were given to prevent or treat conditions for which there is only limited evidence of their efficacy [58].

Our results show that supplement use correlated with “being on a diet”. One explanation may be that the parents attempted to substitute potential nutritional deficits resulting from lower variety of diet. On the other hand, despite high rates of food selectivity and mealtime problems found in our study, we did not observe their correlation with supplement use. The majority of our sample, both with and without ASD, were in preschool age, which is associated with increased occurrence of common childhood illnesses, thus, we hypothesize that part of the individuals were receiving supplements to boost immunity.

Although vitamins and minerals were frequently used both in ASD and control sample, concerning other types of supplements, we found significant differences between the samples. This may result from the fact that supplements are in ASD commonly given as a form of complementary and alternative therapy with the aim to reduce the behavioral symptoms, such as repetitive behavior, to increase social skills, or to improve sleep [51]. In our study, we observed weak negative correlations of supplement use with age and verbal skills indicating that younger and non-verbal children were more likely to use food supplements. In fact, some parents reported that they give the supplements in order to enhance verbal skills in their children presenting delay in language development.

Recently, a meta-analysis revealed that supplementation with vitamins and omega-3 PUFAs had positive effects on some behavioral presentations of subjects with ASD with small effects size [21]. As shown by our results and supported by a study of Trudeau at al. [51], besides vitamins and minerals, the most commonly used supplements included probiotics and omega-3 PUFAs. The administration of probiotics, especially in the context of the “gut–brain-axis”, is a promising intervention for behavioral symptoms, at the same time for GI dysfunction, but clinical studies are still limited and inconclusive [38]. There is evidence that omega-3 PUFAs play role in neurodevelopment, and supplementation with omega-3 PUFAs exhibited positive effects on some core symptoms of ASD. Two meta-analyses gained discordant results, thus more studies are needed to elucidate their efficacy [59]. In our sample with ASD, we also found use of carnosine. Neuroprotective action of carnosine has been reported, and beneficial effects on behavior of subjects with ASD have been observed in some studies, however, they were not confirmed by other studies. At present, there is insufficient support for efficacy of carnosine in ASD [60]. Frequent vitamin D was reported in individuals with ASD [51]. In our ASD sample, 15.0% of supplement users took vitamin D, but we did not include this vitamin into analysis of food supplement use because it is broadly recommended by pediatricians to due to high rates of low plasma vitamin D levels in the population [32].

The effect of food supplements in individuals with ASD appears to depend on the nutritional status, showing that subjects with lower intakes benefit more from the intervention [61]. Still, food supplements are commonly given without previous assessment of nutritional status. The use of food supplements by children and adolescents can improve micronutrient status, but it can also increase the risk of excessive intake of certain vitamins and minerals [57]. According to a study of Stewart et al. [47], despite supplementation, the most common micronutrient deficits in ASD (vitamin D, calcium, potassium, panthothenic acid) were found to have not been corrected. Additionally, the usage of supplement lead to excess intake of vitamin A, folate, and zinc.

Due to a variety of problems associated with ASD, families and caregivers of individuals with ASD face many challenges. GI and nutrition-related problems are often getting less attention in comparison to behavioral or other clinical concerns [3]. There is evidence that GI dysfunction, feeding problems, or imbalanced diet may result in inadequate nutrient intakes in children with ASD, and subsequent nutrition related health consequences [15,44]. At the same time, symptoms of ASD may be exacerbated due to the underlying GI problems [11]. This indicates that multi-professional approach to individuals with ASD should include early identification and management of GI symptoms, food selectivity, and mealtime problems. Currently, no special treatment methods to GI problems specific for subjects with ASD exist and clinical practice guidelines available for the general population should be used [3,62,63]. Concerning food selectivity and mealtime problems, the only empirically supported treatment for children with ASD is behavioral intervention based on applied behavioral analysis (ABA) [45,64,65].

At present time, no causal therapy for ASD core symptoms is known, however, there is scientific evidence about efficiency of behavioral interventions, such as ABA [3]. Despite limited evidence and poor understanding of the potential mechanisms of dietary interventions that might affect behavior, special or elimination diets and nutritional supplementation are frequently used treatments in children and adolescents with ASD [24]. Parents who are considering dietary intervention should contact a health professional (e.g., a pediatrician, or a dietitian), who can provide guidance in planning of the diet to ensure the nutritional needs and monitor the child’s nutritional status.

This is the first study that evaluated GI symptoms, feeding problems in Slovak children and adolescents. The strength of the study is the well-defined sample of children carefully diagnosed for the presence of ASD with golden standard methods, the number of participants, and inclusion of multiple markers into the analysis. The study has its limitations. The sample was not randomly selected, and it included all volunteers who met the criteria, thus, the results may be biased by the sample selection. Another limitation is the fact that data on GI problems were collected by a questionnaire filled in by parents without verification of the diagnosis by a medical doctor. However, according to a study [12], parental report of a GI disorder displayed a high concordance with the clinical diagnosis by a medical professional. Additionally, the subjects in the control sample were not screened for symptoms of ASD, and absence of the disorder was based only on a parental report.

## 5. Conclusions

The present study demonstrated that higher frequency of GI symptoms, food selectivity, and mealtime problems are a common problem in pre-schoolers, schoolchildren, and adolescents with ASD, and together with dietary modification they are significantly associated with ASD. We found that dietary restrictions were introduced also to children exhibiting food selectivity, as well as extensive use of food supplements, although it often lacks sufficient scientific support. From the public health prospective, identification of these problems, their treatment, as well as a need for professional counselling for the caregivers needs to be emphasized. Additionally, GI, nutritional, and feeding problems should be included in education of professionals providing care to individuals with ASD. Higher prevalence of GI problems in girls than in boys with ASD was found, and our results suggest that presence and severity of GI symptoms might be a supplementary biomarker in diagnosing ASD in girls. Many unanswered questions remain in respect to GI, feeding, and dietary issues of children with ASD. This indicates a need for additional studies that will shed more light into these topics.

## Figures and Tables

**Table 1:** Sample characteristics.

Variables	ASD	Controls	*p*
Total number (*n*)	247	267	
Boys	213 (86.2%)	145 (53.2%)	0.000
Girls	34 (13.8%)	125 (46.8%)	
Age (years)			
Range	2–17	2–18	
Median (IQR)	5.4 (3.4–8.6)	5.7 (3.8–8.5)	0.395
Mean ± SD	6.3 ± 3.6	6.5 ± 3.5	0.596
Age groups			
2–5	139 (56.2%)	146 (54.7%)	0.583
6–10	75 (30.4%)	91 (34.1%)	
11–18	33 (13.4%)	30 (11.2%)	

ASD—Autism spectrum disorder; IQR—interquartile range, SD—standard deviation.

**Table 2:** Behavioral characteristics of the participants with autism spectrum disorders (ASD).

Behavioral Characteristics	Values
ADI-R scores (mean, SD)	
Domain A: Qualitative abnormalities in reciprocal social interaction	17.64 (6.23)
Domain B: Qualitative abnormalities in communication	11.98 (4.07)
Domain C: Restricted, repetitive, and stereotyped patterns of behavior	5.37 (2.39)
ADOS-2 calibrated scores (mean, SD)	
Social Affect (SA)	7.29 (1.87)
Restricted and repetitive behavior (RRB)	7.64 (2.18)
Calibrated severity score (CSS)	7.53 (1.75)
ADOS-2 symptom severity (n, %)	
1—mild symptoms (CSS 3–4)	13 (5.3%)
2—moderate symptoms (CSS 5–7)	112 (45.3%)
3—severe symptoms (CSS 8–10)	122 (49.4%)
Spoken language skills (*n*, %)	
Verbal	116 (47%)
Non-verbal	131 (53%)

ADI-R—Autism Diagnostic Interview-Revised; ADOS-2—Autism Diagnostic Observation Schedule—Second Edition; SD—standard deviation; n, %—number, percent of individuals.

**Table 3:** Frequency and types of gastrointestinal (GI) symptoms in children and adolescents with autism spectrum disorders (ASD) and controls (*number*, % of total).

GI Symptoms	ASD	Controls	*p*
Frequency of symptoms			
Never	28 (11.3%)	56 (21.0%)	0.000
1–4 times per month	102 (41.1%)	146 (54.7%)	
Several times per week or daily	118 (47.6%)	65 (24.3%)	
Type of symptoms			
Constipation/hard stools	153 (61.9%)	119 (44.6%)	0.000
Bloating	122 (49.4%)	116 (43.6%)	0.177
Loose stools/diarrhea	115 (46.6%)	119 (44.6%)	0.651
Voluminous stools	126 (51.0%)	59 (22.1%)	0.000
Abdominal pain	77 (31.2%)	104 (39.0%)	0.065
Pain in defecation	50 (20.2%)	42 (15.7%)	0.182
Cumulating of symptoms			
1–2 symptoms	84 (38.3%)	108 (51.2%)	0.002
3–4 symptoms	102 (46.6%)	86 (40.7%)	
5–6 symptoms	33 (15.1%)	17 (10.1%)	

**Table 4:** Frequency of feeding problems in children and adolescents with autism spectrum disorders (ASD) and controls (*n*, % of total sample).

Feeding Problems	ASD	Controls	*p*
Food selectivity			
Never	76 (30.9%)	163 (62.9%)	0.000
1–4 times per month	50 (20.3%)	62 (24.0%)	
Several times per week or daily	120 (48.8%)	34 (13.1%)	
Mealtime problems			
Never	87 (35.7%)	189 (74.1%)	0.000
1–4 times per month	55 (22.5%)	44 (17.3%)	
Several times per week or daily	102 (41.8%)	22 (8.6%)	
Type of mealtime problem			
Anger	130 (53.1%)	67 (25.9%)	0.000
Requests to have food prepared in the same way	104 (42.4%)	72 (27.8%)	0.001
Crying or screaming	50 (20.4%)	7 (6.6%)	0.000
Self-injurious behavior	39 (16.0%)	2 (0.8%)	0.000

**Table 5:** Frequency and types of dietary interventions in children and adolescents with autism spectrum disorders (ASD) and controls (*n*, % of total sample).

Frequency and Types of Dietary Interventions	ASD	Controls	*p*
Dietary intervention			
Yes	52 (21.2%)	13 (4.9%)	0.000
No	193 (78.8%)	253 (95.1%)	
Type of diet			
Gluten and casein (GFCF—gluten free casein free diet)	21 (40.4%)	0 (0.0%)	0.000 *
GAPS diet (gut and psychology syndrome diet)	8 (15.4%)	0 (0.0%)	0.000 *
Gluten free or restricted	9 (17.3%)	2 (15.4%)	0.031 *
Casein free or restricted	3 (5.8%)	2 (15.4%)	0.673 *
Lactose or milk free or restricted	12 (23.1%)	10 (76.9%)	0.663
Restriction/elimination of other foods	16 (30.8%)	3 (23.1%)	0.002 *

* Fisher’s exact test.

**Table 6:** Use of dietary supplements in children and adolescents with autism spectrum disorder (ASD) and controls (*n*, % of total sample).

Use of Dietary Supplements	ASD	Controls	*p*
Yes	165 (66.7%)	172 (64.4%)	0.570
No	82 (33.3%)	95 (35.6%)	
Type of supplements			
Multivitamin/mineral	72 (43.4%)	69 (40.1%)	0.544
Probiotics	71 (42.8%)	55 (32.0%)	0.036
Omega-3 PUFAs	47 (28.3%)	6 (3.5%)	0.000
Carnosine	21 (12.7%)	1 (0.6%)	0.000
Vitamin B	21 (12.7%)	11 (6.4%)	0.050
Vitamin C	18 (10.8%)	70 (40.7%)	0.000
Imunoglukan	13 (7.8%)	33 (19.2%)	0.003
Other	42 (25.3%)	9 (5.2%)	0.000

PUFAs—polyunsaturated fatty acids.

**Table 7:** Multivariate logistic regression analysis of factors associated with autism spectrum disorder (ASD).

Variable	OR	*p*	95% CI Lower	95% CI Upper
Age	0.973	0.419	0.91	1.04
Gender (M = 1, F = 0)	8.263	0.000	4.72	14.5
GI symptoms (range 0–4)	1.337	0.001	1.12	1.60
Diet (1 = yes, 0 = no)	6.342	0.000	2.92	13.8
Supplement use (1 = yes, 0 = no)	0.653	0.092	0.40	1.07
Food selectivity (range 0–4)	1.466	0.000	1.22	1.76
Mealtime problems (range 0–4)	1.591	0.000	1.31	1.93

M—male, F—female, GI—gastrointestinal; OR—odds ratio, CI—confidence interval, deviance goodness of fit chi-square = 465.8 (*p* = 0.768), deviance (likelihood ratio) chi-square = 224.0 (*p* < 0.001).

**Table 8:** Correlation matrix based on pairwise Spearman correlation coefficients (ρ) in the ASD group.

Variable	Age	Gender (1—M, 0—F)	GI Symptoms	Food Selectivity	Mealtime Problems	Dietary Intervention	Food Supplement Use	Domain A (ADI-R)	Domain B (ADI-R)	Domain C (ADI-R)	SA CS (ADOS-2)	RRB CS (ADOS-2)	Total CSS (ADOS-2)	Verbal (1 = Yes, 0 = No)
Age	1													
Gender (1—M, 0—F)	−0.012	1												
GI symptoms	0.070	**−0.225**	1											
Food selectivity	0.105	−0.014	**0.216**	1										
Mealtime problems	0.095	0.009	**0.180**	**0.531**	1									
Dietary intervention	−0.047	−0.088	−0.014	**−0.144**	0.002	1								
Food supplement use	**−0.270**	0.011	−0.020	−0.011	0.051	**0.224**	1							
Domain A (ADI-R)	**0.164**	0.028	**0.164**	**0.158**	0.068	0.048	−0.094	1						
Domain B (ADI-R)	**0.287**	−0.039	**0.133**	**0.165**	**0.178**	−0.024	−0.081	**0.523**	1					
Domain C (ADI-R)	**0.136**	0.102	**0.168**	**0.236**	**0.210**	−0.031	−0.107	**0.413**	**0.323**	1				
SA CS (ADOS-2)	**0.197**	**−0.134**	−0.004	0.107	0.094	−0.050	−0.043	**0.229**	**0.210**	−0.061	1			
RRB CS (ADOS-2)	**−0.320**	0.003	**−0.170**	−0.031	−0.010	−0.021	0.016	−0.031	**−0.165**	−0.025	0.041	1		
Total CSS (ADOS-2)	0.002	−0.080	−0.048	0.047	0.039	0.003	−0.063	**0.178**	0.071	−0.063	**0.774**	**0.480**	1	
Verbal (1 = yes, 0 = no)	**0.499**	−0.076	0.096	**0.150**	**0.139**	**−0.169**	−0.120	**−0.232**	**0.332**	0.054	0.057	**−0.306**	−0.109	1

Values in bold are significant at a 0.05 significance level; ASD—Autism spectrum disorders; M-male; F—female; GI—gastrointestinal; ADI-R—Autism Diagnostic Interview-Revised; ADOS-2—Autism Diagnostic Observation Schedule—Second Edition; SA CS—social affect calibrated score (ADOS-2); RRB CS—restricted and repetitive behavior calibrated score (ADOS-2); Total CSS—Total Calibrated Severity Score (ADOS-2); Domain A—qualitative abnormalities in reciprocal social interaction (ADI-R); Domain B—qualitative abnormalities in communication (ADI-R); Domain C—restricted, repetitive, and stereotyped patterns of behavior (ADI-R). Marked correlations (bold) are significant at *p* < 0.05.
